# Occupation-Based Tele-Intervention for Children with Neurodevelopmental Disorders: A Pilot Study

**DOI:** 10.3390/children12111521

**Published:** 2025-11-10

**Authors:** Stav Ben Zagmi-Averbuch, Deena Rozen, Bathia Aharon-Felsen, Revital Siman Tov, Jeffrey Lowengrub, Miri Tal-Saban, Yafit Gilboa

**Affiliations:** 1 Meuhedet Health Maintenance Organization (HMO), Ashdod 9108601, Israel; deena_rozen@meuhedet.co.il (D.R.); batia.a@meuhedet.co.il (B.A.-F.); simantov_revital@meuhedet.co.il (R.S.T.); lowengrub_j@meuhedet.co.il (J.L.); 2 School of Occupational Therapy, Faculty of Medicine, The Hebrew University of Jerusalem, Jerusalem 9124001, Israel; miri.tal-saban@mail.huji.ac.il (M.T.-S.); yafit.gilboa@mail.huji.ac.il (Y.G.)

**Keywords:** child development, occupational therapy, cognitive orientation to (daily) occupational performance (CO-OP), Tele-CO-OP, tele-intervention

## Abstract

**Highlights:**

**What are the main findings?**

- Tele-CO-OP enables meaningful functional gains in children with NDDs through telehealth.

**What are the implications of the main findings?**

- Home-based delivery enhances the generalization of treatment to the child’s natural environment.
- Key facilitators and barriers identified can inform sustainable teleintervention.

**Abstract:**

**Background:** There is a growing gap between the increasing prevalence of children with neurodevelopmental disorders (NDDs) and the limited availability of developmental services. This raises an urgent need for effective and accessible intervention models. Hybrid intervention offers an innovative and practical solution, yet evidence regarding its feasibility and efficacy for children remains limited. This study aimed to adapt an evidence-based occupational therapy (OT) intervention model for remote delivery and to examine its feasibility and preliminary efficacy among children with NDDs. **Methods**: Using a quasi-experimental pre–post, mixed-methods design, children aged 5–8 years with NDDs were recruited from child development units in southern Israel. The intervention comprised 12–15 weekly video-conference sessions utilizing the Cognitive Orientation to (daily) Occupational Performance (CO-OP) approach. Standardized outcome measures assessed feasibility and preliminary efficacy. Focus groups with parents and therapists explored facilitators and barriers to implementation. **Results**: Of the 26 participants enrolled, 14 children (71% boys) completed the intervention and reported high satisfaction. Clinically significant improvements were observed in personal goal training, reported by both children (80%) and parents (73.68%). Content analysis identified three key themes: parents’ engagement, ecological intervention, and technological literacy. **Conclusions:** Tele-CO-OP intervention was found to be feasible for children with NDDs and showed potential to improve occupational performance in personal goals. Findings provide a practical foundation for developing hybrid OT services as a valuable complement to in-person care for this growing population.

## 1. Introduction

Neurodevelopmental disorders (NDDs) are a group of developmental conditions, including attention deficit hyperactivity disorder (ADHD), autistic spectrum disorders (ASD), and developmental coordination disorder (DCD), characterized by developmental deficits that can be detected during early childhood [1]. They affect 5–20% of the general population and are more frequent in males than females [1]. NDDs are among the most prevalent conditions associated with disability and participation restrictions during childhood. NDDs are also associated with disrupted brain development that affects different aspects of daily life functions [2]. Recent evidence demonstrates, for example, altered resting-state functional connectivity in children with ADHD, reflecting atypical brain network organization [3].

Children with NDDs face significant delays in at least one developmental skill, such as motor, language, behavioral, social, and cognitive development [4]. They also struggle in performing activities, including self-care tasks (e.g., dressing, feeding, and toileting), academic and pre-academic activities (e.g., handwriting, reading, and following classroom routines), and play and leisure activities that require social interaction, motor coordination, or sustained attention [5]. These difficulties lead to functional limitations in daily participation, raising the need for appropriate support and developmental services, sometimes even before the child enters school [6].

NDDs usually continue into adolescence and adulthood, characterized by problems in many areas of function (e.g., work and education), increasing the risks of unemployment, social isolation, injuries, gambling addictions, and delinquency [7]. Lack of early and appropriate intervention can lead to deterioration in overall health and functioning, as well as increase the risk for the emergence of comorbid mental health disorders. Therefore, early intervention among children with NDDs is essential for their long-term health [8].

An intervention approach with strong evidence for improving functioning in children with NDDs is the task-oriented approach [9]. This intervention focuses on a client’s personal goals, empowering them to acquire functional skills and increase participation in their natural environments [10,11]. One example of task-oriented intervention is The Cognitive Orientation to (daily) Occupational Performance [CO-OP; [12]].

The CO-OP approach is a performance-based use of metacognitive strategies and problem-solving principles to improve daily functions [12]. Its aims are to accomplish the following: (a) meet the client’s functional goals through task acquisition; (b) facilitate self-generated cognitive strategy acquisition; and (c) promote generalization and transfer to other occupations and contexts [13]. The key elements of this approach include setting personal functional goals according to the child’s preference; dynamic performance analysis (DPA), using global and specific strategies; and a guided discovery process with enabling principles. The process known as “Goal-Plan-Do-Check” is a global problem-solving strategy that outlines four steps toward achieving goals: setting a functional goal, designing a plan to achieve it, implementing the plan, and checking that it was implemented and that it succeeded. If the plan does not succeed, new plans are generated, and the cycle is repeated [14].

The CO-OP was originally developed to improve functioning and participation in daily activities for children with DCD [13]. Recent evidence has demonstrated the successful application of this approach among children with additional NDDs such as learning disability [LD], ASD, and ADHD [15]. Improvements in goal attainment, activity performance, and transfer to untrained tasks were reported in studies involving children following acquired brain injury (ABI), and in a systematic review of CO-OP interventions with children aged 0–18 years diagnosed with conditions such as DCD and ADHD, it showed evidence for the intervention’s effectiveness [15,16]. Furthermore, evidence from a recent systematic review reinforces the added value of the CO-OP approach compared to other interventions. The review emphasized that the transfer of learned skills is a realistic and attainable outcome across different ages and populations following CO-OP training, with consistently higher levels of transfer observed relative to control interventions. This focus on metacognitive learning, client-driven goal setting, and generalization represents CO-OP’s added value in comparison with traditional top-down or bottom-up interventions, making it particularly relevant for pediatric populations with neurodevelopmental challenges [17,18].

The CO-OP approach is widely used in an in-person format in clinics with children with NDDs [15]. However, access to in-person interventions is often limited due to barriers such as geographical distance, shortage of trained professionals, and long waiting lists, making it difficult for many families to receive timely services using this approach [19]. A possible solution is to provide service through telemedicine platforms to address these issues [20].

Telemedicine is defined as accessibility to health services and clinical information through the use of a variety of technologies [21]. Occupational therapy (OT) tele-intervention is one of the services included in the American Telemedicine Association [ATA] definition [22]. The use of telemedicine services is rapidly increasing in developed countries because it appears to be cost-effective compared to traditional treatment methods, and it offers additional benefits for patients, caregivers, and organizations [23,24]. Key benefits for patients include improved access to services, especially for those living in peripheral areas; the ability to perform intervention in a patient’s natural environment; increased client service availability; and interaction between patients’ home-based caregivers [25,26]. In addition, tele-intervention may improve the caregiver’s sense of confidence and facilitate the application of the clinician’s primary therapy goals [27].

Previous studies have indicated the utility of tele-intervention among children with NDDs, as well as the responsiveness and satisfaction of their families [28], alongside its complexity and barriers [29]. In addition, a few preliminary studies support the effectiveness of tele-interventions for child development, but the evidence is insufficient due to the small sample sizes, very short interventions, and low sensitivity of outcome measures [30]. Furthermore, the value of remote health services for child development has not been sufficiently assimilated among the health professions, including OT [31,32].

Previous researchers have demonstrated the feasibility and effectiveness of the remote CO-OP approach (tele-CO-OP) among diverse populations with chronic health conditions and functional impairments, such as adolescents with spina bifida [33], cancer survivors [34], and adults with acquired brain injuries [35]. However, to the best of our knowledge, the feasibility and efficacy of tele-CO-OP for children with NDDs have not yet been tested.

The rise in pediatric tele-intervention has created new opportunities to deliver occupation-based interventions in children’s natural environments, increasing access and enabling parent-mediated practice [25,36]. Despite growing evidence for tele-intervention feasibility, there remains limited rigorous data on how cognitive-strategy-based approaches, such as CO-OP, function when delivered remotely, and on the roles of family engagement and digital literacy as mediators of outcome [16,37] The tele-CO-OP model adapts CO-OP principles for remote delivery by incorporating simplified, developmentally appropriate mnemonic supports, visual aids, and explicit parent scaffolding to enhance comprehension and strategy application.

Therefore, the goal of this study was to investigate the feasibility, acceptability, and preliminary efficacy of the tele-CO-OP approach among children aged 5–8 years with NDDs. The specific research hypotheses were as follows:

(1) Feasibility: The tele-CO-OP will be found to be applicable in terms of adherence (the number of participants who complete the intervention program, and the number of sessions completed).

(2) Acceptability: The tele-CO-OP will be found to be acceptable to the patients and therapists, as indicated by questionnaires and insights from focus groups.

(3) Preliminary Efficacy: The tele-CO-OP will be found to be measurably effective in improving the function and participation of children with NDDs.

## 2. Materials and Methods

### 2.1. Design

This pilot study employed a mixed-methods, quasi-experimental pre–post design that integrated quantitative and qualitative approaches. The intervention phase served as a preliminary step in preparation for a full-scale study and was complemented by post-intervention focus groups. Mixed-methods designs are commonly used in pilot research to inform the development of larger and more complex studies [38].

### 2.2. Participants

Using a convenience sampling, 26 children and their parents underwent baseline assessments; out of this group, 14 children (71% boys) aged 5–8 (M = 6 years, 5 months, SD = 1.1) and their parents completed the entire intervention. The recruitment was conducted across child development centers in the southern region of Israel. The participants were children who either had a confirmed diagnosis of NDD or were suspected to have an NDD based on professional recommendation. The latter group included children referred by parents or educational staff who had received a developmental evaluation by an occupational therapist, indicating the need for further intervention. Inclusion criteria were as follows: (a) Hebrew at a mother-tongue level; (b) child’s ability to identify at least three functional difficulties, in order to set personal goals; and (c) availability of a computer/iPad with a web camera and wireless connectivity in the child’s home. Exclusion criteria were as follows: (a) children diagnosed with autistic spectrum disorder (ASD) or brain injury; and (b) children with a primary diagnosis of a mental health disorder. These exclusion criteria were defined due to the heterogeneity of functional profiles in these populations and the cognitive–linguistic abilities required for meaningful participation in the CO-OP approach.

Following the pilot study, all the parents and OTs who took part in the study were invited to participate in separate focus groups. Eventually, two focus groups of mothers were conducted (n = 7) and a third focus group that included OTs (n = 5). The OTs were all females, aged between 28 and 50 years, with professional experience ranging from 2 to 24 years.

### 2.3. Measures

#### 2.3.1. Socio-Demographic Questionnaire and Clinical Data

Parents completed a brief sociodemographic questionnaire, providing information about their child’s age, gender, family composition, and educational setting.

#### 2.3.2. Therapist Feasibility Log

The OTs who conducted the intervention documented field notes following each session, regarding the number and duration of the meetings performed. They also reported the use of the key elements in the CO-OP approach to ensure the implementation of its critical components as described in fidelity checklists [39].

#### 2.3.3. Parents as Partners in Intervention—Satisfaction Questionnaire [PAPI-Q; [40]]

This structured questionnaire, developed originally in Hebrew, was designed to assess parental involvement with OT service, such as therapy procedures, session attendance, and general parental satisfaction. In the current study, the parents as well as the OTs were asked to fill out the questionnaire following the intervention to assess the acceptability of the tele-CO-OP. The questionnaire includes 17 items ranked on a 1–5 Likert scale, with a higher score indicating a higher level of satisfaction.

Cronbach’s alpha was good (α = 0.80), suggesting high internal reliability. Content validity was established by a group of 12 OTs. Moreover, the PAPI-Q and the Canadian Occupational Performance Measure (COPM) have been found to have a strong relationship, with a significant positive correlation between parent satisfaction scores, which suggests that both tools are measuring related aspects of the intervention experience [41,42].

#### 2.3.4. Canadian Occupational Performance Measure (COPM), 5th Edition [41]

The COPM is a widely used, valid outcome measure in OT and specifically in developmental services, designed to measure the clients’ self-perceptions of their activity performance, satisfaction with their performance, and changes over time. Participants, in collaboration with their parents, were asked to identify personal and functional goals. Thereafter, they were asked to rate their performance and their satisfaction on a scale of 1 to 10, where 10 indicates optimal performance and/or satisfaction. A clinically significant difference is a minimum of a 2-point change in the COPM performance ratings [41]. Each child completed the COPM performance and satisfaction ratings on an individual form while their parent was present in the same room. The therapist facilitated the process to ensure that children understood the questions and responded independently. Parents completed a separate COPM form reflecting their own perceptions of their child’s performance and satisfaction. Psychometric properties include good predictive validity, which improves therapists’ understanding of clients’ goals and enhances predictive outcome accuracy [43]. Furthermore, it demonstrates good test–retest reliability (0.80 on the performance scale for 1–2 weeks), sensitivity to change in many studies, and usefulness as an outcome measure among various populations, including children [44].

#### 2.3.5. Performance Quality Rating Scale [PQRS; [45]]

The PQRS is an observational measure of performance quality in client-selected, personally meaningful activities that complements the COPM by assessing actual, rather than perceived, performance of the identified goals. The PQRS was originally developed for children with NDDs. The therapist scores each activity pre- and post-intervention, using a 10-point performance rating scale between 1 (“unable to perform”) and 10 (“performs well”). The instrument is commonly used in studies involving intervention according to the CO-OP approach [11]. It has good inter-rater reliability (0.71–0.77) and good test–retest reliability (>0.80). However, convergent validity with the COPM is inconsistent, and further validation is required [46].

### 2.4. Focus Groups

Focus groups were conducted post-intervention to gather in-depth feedback on the tele-CO-OP intervention. Two researchers with experience in facilitating focus groups and qualitative data collection conducted the group sessions. Both were independent of the intervention process to ensure objectivity. Using structured, open-ended questions, parents and therapists were invited to reflect on their overall experience, including factors that facilitated or hindered the process. This approach aimed to evaluate the acceptability of the intervention while also capturing participants’ perspectives to inform future refinement and implementation of the tele-CO-OP model.

Example questions included the following: “Which component of the intervention was most helpful in supporting the process?” (therapists’ focus group protocol) and “If you were aware that two service delivery modes had equal effectiveness, which would you prefer and why?” (parents’ focus group protocol).

### 2.5. Procedure

The research was conducted at the child development centers of Israel’s “Meuhedet” HMO. Ethical authorization was obtained from the Israeli HMO—Meuhedet Health Services (Israel) in 2017 (Ethical code: HMO #05-02-09-20).

Children who applied for OT service were screened for eligibility. Parents provided written informed consent for their child’s participation, and children gave verbal assent in accordance with ethical procedures. Baseline and post-intervention assessment sessions (around 1 h each) were conducted in-person in the clinics by the same OT who provided the intervention. In addition, focus groups were conducted post-intervention with parents and therapists to obtain feedback regarding the tele-CO-OP intervention. They were facilitated by researchers who did not conduct the intervention themselves and were conducted via videoconferencing, recorded, and transcribed.

At the baseline assessment, each participant (with the parent) identified three to five functional goals and rated the importance of each goal using the COPM. Three out of these goals were practiced directly during the intervention (trained goals), while the two remaining goals were untrained in order to allow an examination of the generalization transfer of the strategies learned during the intervention to the other stated goals (untrained goals).

### 2.6. Intervention

The intervention protocol (Appendix A) was based on an adapted remote version of the CO-OP approach [33,35], along with findings from a survey among OTs regarding occupation-based tele-intervention [47]. The protocol included 12–15 weekly video-conference sessions and was based on the CO-OP principles, without any changes in the key elements of the approach [12]. The adjustments mainly involved the therapy setting and environment organization (e.g., choosing the room, gathering required tools, and reducing external stimuli). In addition, electronic tools (such as whiteboards and computerized games) were made available using the Poppins platform (https://femi.com/technology/poppins-3/, accessed on 8 November 2025.), a secure software licensed for use in Meuhedet HMO child development clinics.

The intervention was carried out by eight OTs who were trained by two senior occupational therapists certified by the International Cognitive Approaches Network (ICAN) and who served as study supervisors.

All OTs worked in child development clinics in Israel’s southern district and had at least two years of experience. The OTs were further trained in the use of the research instruments, as well as in the delivery of the treatment protocol. In addition to experience in using the CO-OP approach, OTs underwent additional training that taught them the adjustments needed for remote access.

As part of the first intervention session, the OT and participant reviewed the goals and discussed them; then, the global problem-solving strategy (Goal-Plan-Do-Check) was taught. In subsequent sessions, the OTs guided the participants in using this strategy to discover their performance problems and develop specific strategies in order to improve their performance and accomplish their goals. Examples of domain-specific strategies used during the adapted version of the tele-CO-OP intervention are summarized in Table 1. Rather than providing solutions, the OTs facilitated this process by asking questions and providing feedback.

At the beginning of the intervention, the therapist emphasized the crucial nature of the caregiver’s involvement during the process: according to the CO-OP principles [14], their role is to facilitate the execution of plans and generalizing strategies and skills in daily routines. The parents’ expected involvement was further explained in two categories. First was their role as an agent of change, to encourage their child to acquire problem-solving abilities in various functions and relationships. Second was parental presence during the technical aspects of the treatment, which included assisting with accessibility as needed. Examples included helping the child use technological devices, changing the camera angle when needed, making and sending videos, and organizing the home environment. Occasionally, their supervision was necessary for safety reasons (e.g., cutting food in the kitchen).

Since the CO-OP is a performance-based approach, some of the activities were performed by the participants during the sessions. Whenever possible, the therapist observed those activities performed in the participant’s natural environment via video conferencing. Certain activities could not be performed online, because they required either another setting (e.g., a playground) or privacy (e.g., dressing themselves); the performance strategies and plans for these activities were discussed during online sessions.

In addition, participants received a folder with pictorial aids for use in the sessions. The visual symbols represented the “Goal-Plan-Do-Check” process, in order to make the global strategy more accessible and easier to practice. The visual aids were divided into boys’ and girls’ versions. In some cases, changes were made in the visual aids for cultural adaptation, such as adhering to religious dress codes.

### 2.7. Data Analysis

The data analysis was performed using the SPSS software (Version 25; IBM Corp, Armonk, NY, USA). Descriptions of sample characteristics were analyzed using descriptive statistics. A nonparametric statistical (Wilcoxon) analysis for paired samples was used to assess differences pre- and post-intervention (goals accomplished and changes in quality of activity performance). The significance level for confirming the research hypothesis was *p* < 0.05. Qualitative data were analyzed using a conventional content analysis method [48]. Researchers conducted a reflective process throughout the entire analysis regarding their ideas, perspectives, and thoughts to ensure trustworthy findings. The process included three researchers, all of whom had expertise in child development and remote intervention, and two of them had prior experience in qualitative research, to strengthen the reliability of the findings. One researcher performed the initial open coding of the focus-group transcripts, after which two additional researchers independently reviewed and commented on the emerging codes. The three researchers then met to discuss the coding, refine the categories, and reach full agreement on the final themes.

## 3. Results

Of the 26 children who initially enrolled in the study, 14 completed the full tele-CO-OP intervention (retention rate = 54%), aged 5–8 years (M = 6 years, 5 months; SD = 1.1); the male–female ratio was 10:4. All fourteen children were born in Israel, and their family size ranged from two to eight children (M = 3.86, SD = 1.91). All 14 children studied at regular education programs, and half of them (n = 7) had participated in OT sessions before the current study. The socio-demographic and clinical characteristics of the participants are presented in Table 2.

### 3.1. Feasibility

**Figure 1:**
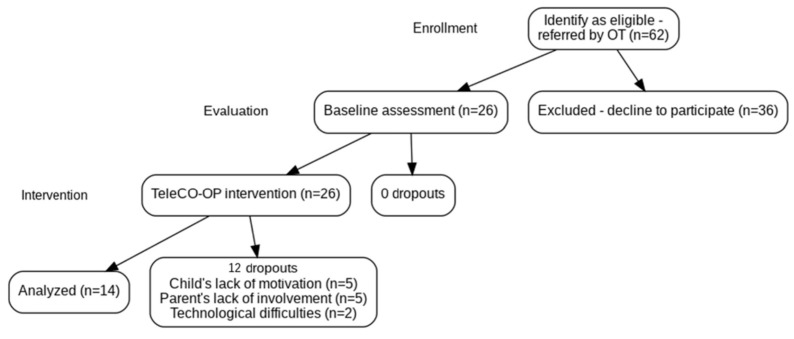
Flow diagram of the study enrollment, evaluation, intervention, and analysis.

A flow diagram provides details regarding the process of recruitment and intervention (see Figure 1). All the children had been referred to one of three child development centers; they were screened for eligibility during a recruitment period of six months. A total of 62 children and their families were found to be suitable and were invited to participate in the study. Of these, 42% (26/62) agreed to participate and started the procedure. Fourteen of the 26 participants completed the 3-month tele-CO-OP program (A retention rate of 54%). Dropouts occurred after 1–6 sessions. The main reasons for discontinuing were as follows: (a) child’s lack of motivation to participate in the intervention (e.g., preference for playing with other toys in the room, limited interest in the treatment activities or conversations, or preference for in-person sessions among children with previous face-to-face therapy experience); (b) parent’s lack of involvement during sessions and training (e.g., being occupied with household tasks or other siblings, or having difficulty practicing and integrating the strategies at home between sessions); and (c) technological issues (e.g., difficulties logging into the platform, unstable internet connection, or poor audio quality).

Participants altogether defined, using the COPM, 64 basic activities from which were derived participation goals (trained and untrained). They covered three main life domains: (a) self-care (n = 16; 25%), for example: eating a meal independently, using a spoon without dropping the food, or getting dressed independently in the morning; (b) productivity in education (n = 40; 62.5%), for example, organizing the required equipment for school, according to the class schedule; and (c) leisure (n = 8; 12.5%), for example, playing chess with a brother, or riding a bicycle independently at the playground. It should be noted that all participants chose at least one education productivity goal.

### 3.2. Acceptability

Acceptability of the intervention was based on a post-intervention satisfaction questionnaire (PAPI-Q). Overall, both parents and OTs expressed high satisfaction with tele-intervention, although the parents reported higher satisfaction. On a scale of 1–5, the average PAPI-Q score was 4.34 ± 0.47 for parents and 4.17 ± 0.53 for occupational therapists (Table 3). Eleven parents (84.6%) and seven therapists (50%) expressed high to very high satisfaction (4–5) with the intervention in general (item 17).

Beyond quantitative satisfaction ratings, a qualitative content analysis was performed to reflect the perspectives of both parents and therapists and to gain insight into their subjective experiences with the intervention process. Three primary themes emerged from the combined analysis of all focus groups. The first theme was *Parents’ engagement*. Participants in all groups indicated that the presence and active participation of parents were necessary conditions for the intervention to succeed. Several parents reported that they learned about their child’s needs this way and felt obligated to remain involved during and between meetings. For example: “Parental involvement was crucial. They need to be involved in every aspect of the session, including documenting, photographing, and listening to what the therapist says.” (Parent 2). A similar perspective was echoed by the occupational therapists in their focus groups, who emphasized that parental presence and trust were essential for both the therapeutic process and its continuation at home. As OT 3 noted, “Relationship with parents was highly meaningful because, without their presence, therapy would not have been possible. In my opinion, the trust of the parent in us, in the therapeutic process, and in its significance in the room to conduct the session and continue the practice at home was fundamental.”

The second theme was ecological intervention. Developmental treatments are often performed in clinics, which simulate “laboratory conditions”. Thus, remote treatment in the participants’ homes provided a new therapeutic environment. It was noted positively by many therapists that the tele-intervention enables working on goals that are relevant to daily activities using tools and methods the child is familiar with. As OT 2 described, “I strongly believe in it (therapy in the natural environment) because, in the clinic, there are controlled conditions. However, being in their home, it allowed us to see the family in their own space. We could understand their living conditions, explain and adapt the environment based on their changing needs.” At the same time, therapists described difficulties in delivering the intervention in the home environment, such as coping with distractions and having no control over the treatment conditions. OT 3 explained that, “for some, distractions occur, such as siblings entering and leaving the room, wanting to show things, or being in different parts of the house. Thus, therapy becomes dynamic and unpredictable. As a therapist, you become limited beyond the screen, posing a significant challenge.” Parents, however, also described the home environment as an opportunity for meaningful preparation and adaptation. For instance, Parent 2 noted that “As part of our preparation, we organized our environment. Our therapist asked us to see our home first, explaining how adjustments could be made. She helped us understand how to create the optimal environment for our child. Additionally, we were asked to prepare specific materials before each session. It wasn’t complicated, and we came prepared.”

The third theme was technological literacy. Infrastructure and technological platforms were mentioned by participants with differing opinions. Some reported that the platform served the therapist and the patient’s needs. Parent 4 noted that, “It (the software) was very user-friendly, and we did not encounter many issues. There were games and interesting tools included in the software that my child eagerly looked forward to”. While others found it difficult to learn, use, and operate the technology. Apart from platform type, many OTs mentioned the need to gain more in-depth knowledge, understanding, and proficiency with technological tools. For example, one person said that, “It was challenging to conduct the session many times because the technology didn’t work as it should. Even a slight interruption in the internet made it challenging. I found it to be extremely challenging.” (OT 4).

### 3.3. Preliminary Efficacy

Changes before and after the intervention were measured by the COPM and were analyzed using the Wilcoxon signed-rank test, including effect sizes. The results are shown in Table 4. Significant improvement was found in performance and satisfaction ratings reported by the child and the parent, for both trained and untrained goals. Further analysis of the results demonstrated a clinically significant improvement (≥2 points) in the COPM performance scale relating to 80% and 73.68% of the individual goals practiced during treatment, as reported by children and parents, respectively.

Similar results were also found on the COPM satisfaction scale, which showed clinically significant improvement in 85% of the goals practiced, according to the parents’ reports. Children’s responses on the satisfaction scale were not analyzed due to their consistent difficulty in distinguishing between the scales of the COPM. In addition, another clinically significant improvement (≥2 points) was found on the PQRS scale between pre- and post-intervention, with large effect sizes (see Table 4).

The sample size required for a future study was calculated based on the results of the current (pilot) study we recently conducted. In order to estimate sample size, we relied on the largest conservative effect size found in our primary outcome results (COPM; ES = 0.88) with a two-sided significance level of 0.05 and power of 80% with equal allocation to two arms. According to this calculation, a minimum sample size of 22 participants in each group is required. Following the results of the pilot study, we expect a dropout rate of about 20%; therefore, we decided to recruit 27 participants for each study group.

## 4. Discussion

The current study assessed the feasibility, acceptability, preliminary efficacy, and implementation barriers and facilitators of the CO-OP approach in a tele-intervention format for children with NDDs. Our findings indicated that tele-CO-OP was feasible and acceptable among parents and OTs. In addition, the study provided preliminary evidence for the intervention’s efficacy, as demonstrated by significant clinical improvements in both trained and untrained functional goals. Furthermore, the qualitative analyses of the focus groups revealed three overarching themes that informed meaningful refinements and adaptations to the intervention.

### 4.1. Feasibility

In terms of adherence, 54% of the participants completed all sessions of the tele-CO-OP as required by the research protocol. The retention rate in the current study compares favorably to those reported in a systematic review of 23 tele-intervention studies conducted by healthcare providers among children with disabilities, where the highest reported retention rate was 40% [49]. Despite being consistent with previous reports, these findings highlight the ongoing need for future research to better understand and improve adherence in pediatric tele-interventions.

### 4.2. Acceptability

High levels of satisfaction were reported by the parents, demonstrating the acceptability of the protocol. These findings are consistent with previous studies. For example, a survey found that the majority (80.5%) of parents or caregivers of children with NDDs were satisfied with tele-interventions conducted by health professionals during the COVID-19 pandemic [50]. Even though therapists were generally satisfied with tele-interventions, their satisfaction levels were slightly lower than those of parents. Previous studies have found that the major factor affecting therapists’ satisfaction and confidence with remote services is their previous experience [51,52]. Therefore, the small gap in our study can be explained by the fact that they were first-time users of a tele-intervention [53]. In addition, the lack of a therapist’s previous experience with tele-interventions in the present study might also influence the dropout rate. These findings highlight the need for future research to further explore the relationship between therapists’ prior experience, confidence, and engagement in tele-intervention, as well as the types of support or training that may enhance their satisfaction and sustain participation over time.

The implementation of the tele-CO-OP protocol revealed several key facilitators and barriers that emerged from the focus groups, many of which align with findings from the recent literature [12,29,54,55]. One notable facilitator was active parental engagement, which was identified in our focus groups as a critical component for successful intervention. As mentioned in other studies, pediatric tele-interventions are most effective when clients and their primary caregivers (usually parents) are empowered and guided remotely by OTs [54]. Implementing tele-intervention, as was performed in this study, includes parental and child involvement (a key recommendation in the CO-OP approach) [12]. Similar findings were reported by Steinberg et al. [29], who found that parents who embraced a more central role in remote sessions contributed significantly to reconstructing therapeutic conditions at home. Furthermore, low parental involvement emerged as a barrier, which is consistent with a systematic review by Jimenez-Arberas et al. [55], who found that reduced caregiver availability was associated with lower fidelity and completion rates in tele-intervention.

Another strong facilitator identified in our study was the ecological validity of conducting therapy in the child’s natural environment. Therapists emphasized that delivering sessions at home enabled them to address authentic, everyday challenges using familiar materials and routines. This finding is consistent with Steinberg et al. [29], who, in their qualitative study of telehealth in pediatric rehabilitation, described “home as a new therapeutic opportunity,” highlighting how the home environment can expand the relevance and meaningfulness of intervention. At the same time, therapists in our study also noted practical challenges, including limited physical space, background noise, and competing household stimuli, all of which disrupted the traditional structure of therapy and occasionally hindered session flow. Similar limitations were reported by Buitrago et al. [56], who found that household distractions and privacy concerns require careful planning and adaptation in order to optimize remote intervention.

A third influential factor was technological literacy. While some participants adapted easily to digital platforms, others, especially therapists new to tele-intervention, expressed discomfort or a lack of confidence. Similar concerns were widely reported in the literature, with therapists frequently describing technology-related stress as limiting their ability to focus on therapeutic content [29]. These insights support calls for formal training and digital competence development as a prerequisite for effective implementation.

Taken together, these findings underscore the importance of thorough pre-intervention preparation. This includes providing parents or primary caregivers with an initial orientation, clear guidance, and setting their expectations regarding their role during and between sessions. In addition, technical training on the tele-intervention platform should be offered to both parents and therapists, accompanied by ongoing technical support throughout the intervention process. Regarding the home environment, preparatory steps should focus on organizing the physical space, anticipating and managing potential distractions, and ensuring the availability of appropriate equipment and environmental adaptations.

Integrating these elements may enhance a future implementation’s fidelity and sustainability of tele-intervention services in pediatric rehabilitation.

### 4.3. Preliminary Efficacy

Improving daily activity performance and participation is a highly desired outcome in pediatric OT intervention [57]. Accordingly, the primary outcome in our study was perceived performance change and satisfaction relating to participant-chosen functional goals. Despite the small sample, significant statistical and clinical improvements were found in individual functional goals post-intervention. These results are in line with previous systematic reviews and studies that evaluated the efficacy of tele-interventions among children with a variety of disabilities [32,37,58,59,60]. Our results are also in line with previous studies that evaluated the efficacy of the traditional in-person CO-OP approach among children with NDDs, as well as the use of tele-CO-OP among other populations [34,35]. Capistran and Martini’s study [61], for instance, found similar percentages of goals in which children reported a significant improvement according to the COPM.

In the current study, gains were found in untrained goals, as reported by both parents and children. Our results were similar to those in previous CO-OP studies among children, showing significant improvements not only in trained goals but also suggesting transfer to untrained activities and everyday contexts [62,63]. This confirms that the principles of the CO-OP were designed to allow generalization of the results and transfer to other performance challenges, specifically the metacognitive nature of the approach and the emphasis on generalization and transfer to the child’s natural environment during the sessions. Consequently, participants will be able to use global and domain-specific strategies on their own in a broad range of situations [64].

### 4.4. Limitations and Future Study

Study limitations should be taken into account when interpreting the results. First, these analyses were exploratory, and the sample size was small and relatively homogeneous, as participants were recruited from a specific region in the country. Furthermore, this was a pilot study without a control group, so we cannot necessarily attribute the improvements exclusively to the intervention. Nonetheless, since no other OT service was provided during the intervention period, we can assume that the improvement in activity performance was caused at least in part by the intervention being evaluated. In future studies, we recommend including a larger and more diverse sample, as well as a control group, to strengthen the validity of the findings and confirm this assumption.

Additionally, we included a heterogeneous group of participants with NDDs who had different functional difficulties, while excluding children with ASD, brain injury, and a primary diagnosis of mental health disorders. However, we recommend that future studies change the inclusion criteria to be based on functional profile rather than on etiology.

A further limitation of this study relates to the research measures employed. All outcome measures were subjective in nature, relying on the perspectives and self-reports of the children and their parents. Moreover, these evaluations were administered by the same therapists who delivered the intervention, which may have introduced potential bias. To address these limitations, future studies should incorporate objective assessment tools capable of capturing specific changes in body functions (e.g., executive functions) as a result of the intervention. In addition, outcome evaluations should be conducted by independent therapists, separate from those delivering the intervention, to minimize the risk of bias and strengthen the validity of the findings.

## 5. Conclusions

Based on the findings gleaned from the pilot study, tele-CO-OP for children with NDDs appears to be feasible and has potential efficacy in improving occupational performance to attain participants’ functional goals. Our study contributes to the growing body of evidence supporting the use of tele-intervention as a valuable supplement to in-person interventions, overcoming accessibility barriers and the limited availability of services in remote areas. However, issues have arisen during the focus groups that require attention and adjustment for applicability and scalability. Consequently, a specialized panel was convened for expert discourse, leading to modifications to the protocol, such as the following: (1) The necessity for promoting parental involvement in the therapeutic process; (2) the incorporation of occupational performance coaching [OPC; [65]] as the foundation of the intervention; and (3) adding the parenting sense of competence [PSOC; [66]] as an outcome measure.

In the long run, it can be assumed that integration of accessible and effective services in child development centers will help reduce the burden on families and promote their functioning. Building on these findings, future research can now be conducted through randomized controlled trials with adequately powered sample sizes to rigorously establish the effectiveness of the tele-CO-OP approach.

## Figures and Tables

**Table 1:** Domain-specific strategies (DSS) used to promote goal achievement.

Goal	CO-OP Strategy Use	Example
Organizing the required equipment for school according to the class schedule	Attention to doing	Therapist: How will you remember the required equipment for each day? Client: I will write the schedule on my board inside my room.
	Task specification\modification	Therapist: Where should you place your books? Client: I can reach the lower shelf above the desk by myself.
	Verbal-motor mnemonic	Therapist: How can we call the required stages of this activity? Client: Take out, choose, put in and checking.
Complete writing assignment independently	Body position	Therapist: (demonstrate sitting bent over and away from the table) What do you think about my sitting posture? Client: Your back will hurt. Therapist: What should I do? Client: Approach the table and straighten your back.
	Task specification\modification	Therapist: Which place will allow you enough space to study with no other distractions? Client: My brother and my room, near the desk with my study equipment, while my brother is in the playroom.
	Verbal-motor mnemonic	Therapist: (displays a group of letters) What do all these letters have in common? Client: They are all written in the same direction. A balanced line from above and going straight down. Therapist: Great. What do you think will be a proper name for this group? Client: The ceiling and the wall.
Eating a meal independently using a spoon without dropping the food	Attention to doing	Therapist: Demonstrate eating while standing and watching television. Client: You are spilling the food everywhere Therapist: I didn’t notice, what should I do differently? Client: Your mouth is very far from the plate. Maybe you should sit down and watch the food instead the TV.
	Task specification\modification	An observation on mealtime was performed and recommendations were given by the occupational therapist, such as the following: replace the chair (higher one), and use a shorter spoon and a bowl instead of a flat plate.
	Verbal–rote script	Therapist: How can we remember the sequence of all the steps we need to do? Client: Sit, closer, pick a little, and directly into the mouth.

**Table 2:** Socio-demographic and clinical characteristics of the participants (N = 14).

Characteristics	N (%)	Median (IQR)
Age		6.66 (6.04–7.81)
Gender		
Girls	10 (71.43)	
Boys	4 (28.57)	
Fathers		
Age (years:months)		38:6 (32–42)
Education (years)		15 (12–16)
Mothers (years)		
Age (years:months)		39:6 (35–45)
Education (years)		16 (15–16)
Number of children in the family		3 (2.75–5.25)
Native language		
Hebrew	10 (71.43)	
Bilingual	4 (28.57)	

**Table 3:** Descriptive statistics of parents’ and therapists’ satisfaction with the tele-CO-OP.

PAPI-Q Statements	Parents M (SD)	Occupational Therapists M (SD)
Did the number of sessions match the child’s needs?	4.38 (0.65)	4.43 (0.51)
Did you feel like a partner in the process?	4.54 (0.66)	4.82 (0.40)
Did the child participate in most of the sessions?	4.77 (0.60)	4.64 (0.74)
Did you/the child and his parent practice at home according to the guidance?	4 (0.58)	3.69 (1.18)
In your opinion, did you/the parent acquire tools to implement with other difficulties?	4 (1.00)	3.89 (0.60)
General satisfaction with the therapeutic process.	4.38 (0.77)	3.71 (0.99)
Satisfaction with the topics the therapy dealt with.	4.36 (0.46)	4.08 (0.76)
Total	4.34 (0.47)	4.17 (0.53)

**Table 4:** Changes over time from baseline to post-intervention in the COPM and PQRS.

Measures	Pre Median (IQR)	Post Median (IQR)	Z	P	r (ES)
COPM—performance					
Child (trained goals)	5 (3.29–6.08)	9 (8.1–9.75)	−3.279	0.001	−0.881
Child (untrained goals) ^a^	5 (4–6)	8.5 (7.5–10)	−2.937	0.003	−0.885
Parent (trained goals)	4.83 (3.83–5.67)	(7.17–8.69)	−3.062	0.002	−0.818
Parent (untrained goals) ^a^	6.5 (5.5–7)	8.5 (6.5–9)	−2.392	0.017	−0.721
COPM—satisfaction					
Parent (trained goals)	5 (3.08–5.81)	9.17 (8.1–10)	−3.185	0.001	−0.851
Parent (untrained goals) ^a^	6.5 (4.5–6.5)	9 (8–10)	−2.606	0.009	−0.786
PQRS (trained goals)	4.58 (3.25–5.5)	8 (7.67–8.54)	−3.298	0.001	0.881

Notes. An effect size (r) was calculated from the z value of the Wilcoxon signed-rank test (r = z/√n) and can be interpreted as a small (r ≤ 0.10), medium (r = 0.30), or large (r ≥ 0.50) effect size. ^a^ n = 11.

## Data Availability

Data is contained within the article.
